# Survival and development of *Lycorma delicatula* (Hemiptera: Fulgoridae) on common secondary host plants differ by life stage under controlled conditions

**DOI:** 10.3389/finsc.2023.1134070

**Published:** 2023-02-10

**Authors:** Johanna E. Elsensohn, Laura J. Nixon, Julie Urban, Sharon K. Jones, Tracy C. Leskey

**Affiliations:** ^1^ Appalachian Fruit Research Station, USDA - ARS, Kearneysville, WV, United States; ^2^ Department of Entomology, Pennsylvania State University, University Park, PA, United States

**Keywords:** spotted lanternfly, *Vitis vinfera* L., *Juglans nigra*, greenhouse, specialty crop

## Abstract

Host range assessment for emerging invasive insects is a vital step toward fully defining the issues the insect may pose. Spotted lanternfly (SLF) is an invasive species that is rapidly expanding its presence in the United States. The primary hosts facilitating this spread are tree of heaven, a plant from SLF’s native range, and the economically important winegrape. Black walnut is also implicated as an important and common host plant. This study investigated the survival and development of SLF on diets that included a variety of crop host plants in the presence or absence of tree of heaven. The following plant species, ‘Honeycrisp’ apple, ‘Reliance’ peach, silver maple, and tree of heaven were paired with winegrape or black walnut throughout the study. SLF had strong development and high survival on a diet of winegrape alone, and winegrape or black walnut paired with tree of heaven. Survival parameters were reduced with all other plant pairings. In particular, SLF in the winegrape and peach diet treatment did not develop past the third nymphal instar. A second experiment evaluated the survival of early and late instar nymphs and adult SLF life stages on three specialty crops – ‘Cascade’ hops, muscadine grapes, and kiwifruit over a two-week period. Nymphs survived longer than adults, with survival of first and second instar nymphs on hops not differing from the control tree of heaven treatment. The adult stage survived best on kiwi and muscadine grape. Our results show tree of heaven and winegrape were the only single plant diets evaluated that are sufficient for complete SLF development, while other host plants may require additional host or hosts of sufficient nutritional quality for SLF survival.

## Introduction

Once a novel invasive species becomes established in a new area, factors affecting spread into the surrounding landscape become especially salient. For polyphagous insects, available host plants can be abundant in many ecosystems, while host preference and usage patterns within these ecosystems can appear variable and abstruse. Spotted lanternfly (SLF), *Lycorma delicatula* (White) (Heteroptera: Fulgoridae), is a polyphagous phloem-feeding species established in the USA starting in Berks County, PA (1) with confirmed populations now in 14 states (2). Spotted lanternfly damage – which involves effects from direct feeding, such as loss of vigor, stem dieback, and indirect damage from honeydew excretion causing decreased photosynthetic ability from sooty mold growth (3) – is of great concern for specialty crop growers. At highest risk for economic damage are winegrapes (*Vitis vinifera* L. (Vitales: Vitaceae)), used in the production of wine, raisins, and grapeseed oil (4). Reports from China, Korea, and Pennsylvania reveal SLF damage to additional fruit, vegetable, and tree nut crops (5–7). As risk to susceptible crops from invasive species can be regionally specific due to local biotic and abiotic conditions, it is important to understand host use patterns in each invaded region.

While SLF can fully develop and reproduce on tree of heaven, *Ailanthus altissima* (Mill.) Swingle (Sapindales: Simaroubaceae) (8), SLF fitness feeding on other plant species is more complex. Without tree of heaven, SLF can develop to adulthood on select diets comprised of a single host plant species, though overall fitness is greater when multiple plant species are available (9–11) including tree of heaven (11, 12). Molecular gut content analyses show SLF feed on a variety of species throughout their development (13–15). Together, these results suggest SLF visit multiple hosts to optimize their development and gather necessary nutrients that may be absent from their preferred host, tree of heaven, or that they require multiple plant species to attain adequate nutrition for survival and development (9). In the field, SLF are observed on dozens of plant species throughout their development (16–18). Spotted lanternfly are thought to have their broadest host range during the 1^st^ instar stage, with this range becoming increasingly narrower as it molts into later life stages. Spotted lanternfly nymphs and adults are found on vine and tree species common throughout Eastern US forests (7, 17, 19).

Plant species with vine growth habits usually contain a mixture of woody and herbaceous tissue. As such, all SLF life stages can exploit the various plant parts to access phloem. All SLF stages have been observed feeding on winegrape and poison ivy (*Toxicodendron radicans* (L.) Kuntze (Sapindales: Anacardiaceae)) while other vine species are observed as a feeding source for 1^st^ and 2^nd^ instars only despite yearlong availability (7). Additional vine species grown as specialty crops, such as cucumber, muscadine grape, hop, and kiwifruit, are reported as SLF hosts, though SLF’s utilization of these species in the United States is unclear (7, 11, 20, 21). However, SLF are considered pests of kiwifruit (*Actinidia chinensis* Planch. (Ericales: Actinidiaceae), *Act. deliciosa* (A. Chev.) C. F. Liang & A. R. Ferguson (Ericales: Actinidiaceae)) in China and Korea (22–25).

Risk of SLF inflicting economic damage in US orchard crops is of concern (26), though their pest potential for most crops including orchards remains understudied (27). Spotted lanternfly are a reported pest of apple in China (Xiao 1992, Zhang 1993), however Lee et al. (28) reported SLF were not able to enter the phloem phase of neither apple nor peach plant tissue *via* EPG and showed low survival of nymphs and adults on these hosts. Nevertheless, high populations of SLF adults have been observed in and around US orchards (29, 30). Further research to clarify their host status is warranted, especially in the context of mixed host diets.

The goal of this study was to investigate the potential for SLF to utilize and develop on single and mixed diets of cultivated specialty crop and wild host plants. We quantified SLF survival on cultivated woody vine hosts over a two-week period for early and late nymphal instars and adults. We also used winegrape and black walnut as the primary hosts to assess survivorship and development as they are commonly encountered species in SLF’s current geographic range. Results from this study will add to our understanding on SLF host use and nutritional requirements of each life stage.

## Materials and methods

### Two-week survival on specialty crops

Three crop plants were evaluated as single diets for SLF: ‘Cascade’ hops, *Humulus lupulus* L. (Rosales: Cannabaceae) (Great Lakes Hops); muscadine winegrape, *Vitis rotundifolia* Michx. var. Carlos (Vitales: Vitaceae) (Willis Orchard, Catersville, GA); and kiwifruit, *Actinidia* sp. (Ericales: Actinidiaceae) (grown at Appalachian Fruit Research Station (AFRS)). For kiwifruit, *Act. deliciosa* ‘Hayward’ was grafted onto seedlings of *Act. chinensis* ‘Tango’ (PP32,617) and pollinated by *Act. chinensis* ‘Hombre’. Tree of heaven was used as a control. Tree of heaven plants were grown from field-collected samaras, which had been stratified in a refrigerator at 5 – 7°C for two months. Prior to planting, wings were removed and the remaining seeds from the samaras were soaked in water for 18 h. Seeds were then planted in a tray and placed in an environmental chamber (25°C, 16:8 L:D) to germinate. Once seedlings leafed out, they were transplanted to 0.6 L pots and moved to the greenhouse for maintenance. Healthy trees were then transplanted into 2.7 or 6.5 L pots. All plants for experimental use were maintained in a greenhouse at the AFRS, USDA-ARS, in Kearneysville, WV at a height of ~50 cm (8, 11). At the start of each trial, plants were transported to a quarantine greenhouse at Fort Detrick, MD and placed in a cage (W32.5 x D32.5 x H77.0 cm, 680 µm aperture mesh, BugDorm-4S3074 Insect Rearing Cage, MegaView Science Co., Taiwan). Each cage housed a single host plant in a 6.5 L pot with a water saucer underneath. An 18 L mesh bag covered the saucer and pot and was secured around the base of the plant with a zip-tie to prevent SLF from falling into the water pool. Fifty early instar nymphs (1^st^ and 2^nd^ instars), twenty-five late instar nymphs (3^rd^ and 4^th^ instars), or ten pre-reproductive or reproductively mature adult SLF were introduced into each cage using individuals collected directly from Winchester, VA (APHIS permits P562P-18-03369, P526P-21-04099). Early instar trials were conducted in June and July 2020 (10 – 32°C, average temperature: 20.3°C, 41 – 95% RH, average RH: 59.4%) and May 2021 (17 – 30°C, average temperature: 21.3°C, 22 – 92% RH, average RH: 55.2%); late instar trials were conducted July and August 2020 (10 – 32°C, average temperature: 18.7°C, 40 – 78% RH, average RH: 57.2%) and July 2021 (17 – 33°C, average temperature: 22.2°C, 44 – 98% RH, average RH: 67.5%); pre-reproductive adult trials were conducted September 2020 (6 – 34°C, average temperature: 16.3°C, 35 – 90% RH, average RH: 62.0%), August 2021 (17 – 33°C, average temperature: 21.8°C, 45 – 100% RH, average RH: 73.1%) and September 2021 (16 – 32°C, average temperature: 20.4°C, 47 – 100% RH, average RH: 75.2%); reproductively mature adult trials were conducted beginning in mid-October 2021 (16 – 35°C, average temperature: 19.4°C, 26 – 98% RH, average RH: 61.8%).

All trials were conducted with natural daylengths. Insects were observed for 14 days, during this time the number of dead SLF was recorded and removed every 2 – 4 days. After day 14, the number of surviving SLF was confirmed. Six cages of early instars (total N = 300), six cages of late instars (N = 150), five cages of pre-reproductive adults (N = 50), and three cages of reproductively mature adults (N = 30) were evaluated for each host. Differences in survival distribution within each life stage were assessed using Kaplan-Meier with log-rank (Mantel-Cox) tests for pairwise comparisons (α = 0.05) using the Bonferroni adjustment for multiple comparisons (31) and Cox proportional hazard ratios (HR) to assess instantaneous risk of death. All tests were conducted in R Statistical Software (v2.4.2; 32) using the base, *survival* (33) and *survminer* (34) packages.

### Survival and development on winegrape and black walnut supported diets

The following plants were maintained at 30-50 cm in height in 2.7 L pots for use in single and mixed plant species diet trials: cultivated winegrape, *Vitis vinifera* L. var. Riesling (Amberg Winegrapes LLC, Clifton Springs, NY); black walnut, *Juglans nigra* L. (Fagales: Juglandaceae) (Cold Stream Farm, Free Soil, MI); apple, *Malus domestica* Borkhausen (Rosales: Rosaceae) var. Premium Honeycrisp (Adams County Nursery, Aspers, PA); peach, *Prunus persica* (L.) Batsch (Rosales: Rosaceae), var. Reliance (Dave Wilson Nursery, Hickman, CA); and silver maple, *Acer saccharinum* L. (Sapindales: Sapindaceae) (Cold Stream Farm, Free Soil, MI). Tree of heaven was grown as previously described (8, 11) and maintained in 2.7 L pots at 30 cm height.

Spotted lanternfly egg masses were collected by removal from trees in the field (Winchester, VA) in the winter (Jan/Feb). Egg masses were held in ventilated storage at ambient conditions for 4-8 weeks, brought to the quarantine facility and held in a growth chamber at 10°C until brought into the greenhouse for hatching. Thirty neonate SLF 1^st^ instar nymphs (<48 h old) were introduced into a cage (W32.5 x D32.5 x H77.0 cm, 680 µm aperture mesh, BugDorm-4S3074 Insect Rearing Cage, MegaView Science Co., Taiwan) containing two potted plants. Experimental diets evaluated were: 1) winegrape/winegrape; 2) winegrape/apple; 3) winegrape/peach; 4) winegrape/silver maple; 5) winegrape/tree of heaven; 6) winegrape/black walnut; 7) black walnut/black walnut; 8) black walnut/apple; 9) black walnut/peach; 10) black walnut/silver maple; and 11) black walnut/tree of heaven. Each treatment was replicated three times. All cages were started as neonates emerged, between 1^st^ and 29^th^ April 2021, and held in the greenhouse under natural daylength. Plants were replaced as necessary based on a subjective evaluation of plant health, including the amount of honeydew, presence of yellow and dropped leaves or visible microbial growth. We replaced plant on average every three weeks for 1^st^ – 3^rd^ instars and every 2 weeks when 4^th^ instars and adults were present. Survivorship and development were recorded three times per week until all individuals in a cage died. Development was assessed by visual counts of live individuals and collection of dead insects and nymphal molts. A combination of molts and body size was used to determine the life stage of each insect as they progressed through nymphal instar stages.

When found dead, adult females were collected into 95% ethanol and stored at -20°C. For dissections, legs and wings were removed from specimens, and specimens were imaged and dissected using an Amscope SM-3T stereo microscope and camera. The lateral and ventral aspects of all specimens’ abdomens were imaged to capture the yellow area showing in these regions, which increases as SLF females reproductively mature (30). Imaged specimens were stored in 95% ethanol at room temperature until dissection. Because specimens were desiccated and showed some degradation from exposure prior to initial collection, they were then soaked in a mixture of 200 µl glycerol with 1000 µl 1× Dulbecco’s phosphate-buffered saline solution at room temperature for 24 h prior to dissection. Ovary development was rated using a modified scale based on Nixon et al. (11), such that females were rated as: (1) previtellogenic-I (0-1 immature oocytes/ovarioles detectable; ovaries undeveloped, bright white in color); (2) previtellogenic-II (>1 immature oocytes/ovarioles detectable; bright white in color); (3) vitellogenic-III (ovaries more developed, multiple oocytes on ‘string’; beige to yellowish in color); (4) vitellogenic-IV (ovaries contain many eggs; eggs not fully yellowed and not full size, without hardened/thicken surface); (5) postvitellogenic (eggs filled with yellow yolk; surface hardened. Specimens were also examined for any evidence of having been mated (i.e., for whole or pieces of a spermatophore). Bursa copulatrix development was scored as follows: (I) undeveloped, thin exterior wall; (II) somewhat developed, exterior wall somewhat thickened; (III) features of (II) plus a honeycomb structure visible on wall; (IV) features of (III) plus crystals apparent inside. Bursa copulatrix sclerotization was scored as follows: (I) No sclerotization; (II) minor sclerotization, tan or light brown; (III) highly sclerotized, dark brown; (IV) highly sclerotized with black marks present. Survivorship was analyzed using a Kaplan–Meier analysis with pairwise comparisons (α = 0.05) using the Bonferroni adjustment for multiple comparisons. Development times of each life stage were compared using ANOVA with Tukey’s HSD (honestly significant difference) for mean separation. Analysis was conducted using the base, *survival* (33) and *survminer* (34) R packages (32).

## Results

### Two-week survival on specialty crops

**Figure 1:**
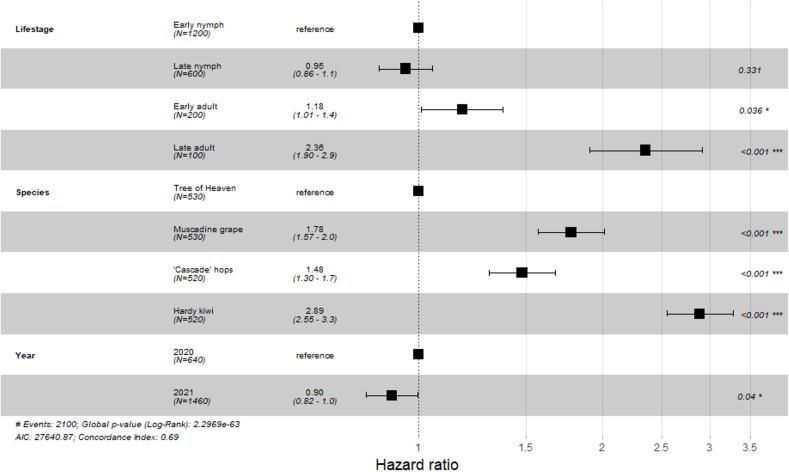
Hazard ratio values for 2-week survival study Each variable within a factor are compared to a reference variable (assigned a value of 1.0). Hazard ratios below 1 indicate a decreased risk of death, while values greater than 1 suggest an increased risk of death as compared to the selected reference. Horizontal bars indicate the 95% confidence interval. Numbers on the right side of the figure are the p-values for each sub-variable, with asterisks indicating the degree of significance: * < 0.05, ** < 0.01, *** < 0.001.

Overall, nymphal SLF had a lower risk of death when feeding on any single host plant compared with adult SLF, with reproductively mature adults having the greatest risk of death (**Figure 1**; Early nymph: HR = 1, late nymph: HR (CI) = 0.95 (0.86-1.05) p = 0.331; pre-reproductive adults: HR (CI) = 1.18 (1.01-1.37), p = 0.036; reproductively mature adults HR (CI) = 2.36 (1.9-2.91), p < 0.001). By host plant, tree of heaven as a feeding host held the lowest chance for death for all SLF life stages, followed by, in order, hops, muscadine grape, and kiwi (**Figure 1**; tree of heaven: HR = 1; ‘Cascade’ hops: HR (CI) = 1.48 (1.3-1.7), p < 0.001; muscadine grape: 1.78 (1.57-2.0, p < 0.001; kiwi: HR (CI) = 2.89 (2.55-3.3), p < 0.001).

**Figure 2:**
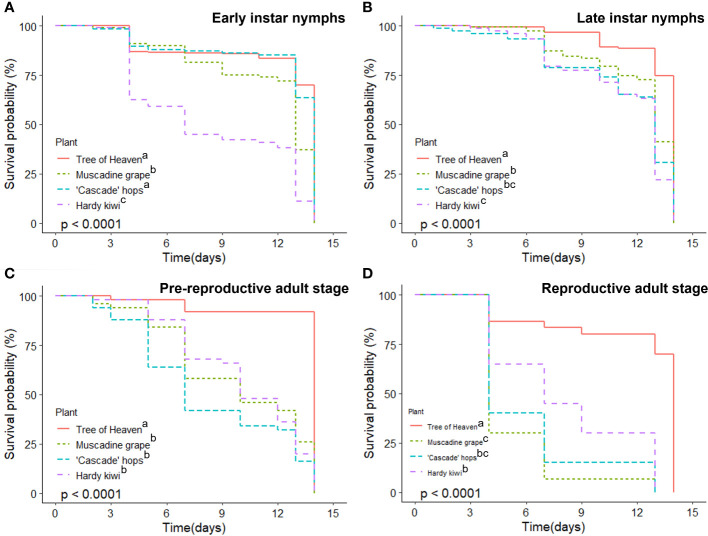
Survival of *L. delicatula* at different development stages for 2 weeks on 4 host species. **(A)** Early nymphs (1^st^ and 2^nd^ instars); **(B)** late stage nymphs (3^rd^ and 4^th^ instars); **(C)** pre-reproductive adults (early September); and **(D)** reproductively mature adults (mid-October). Within each panel legend, plants sharing the same letter after their name are not significantly different from one another at α=0.05.

Survival probability of early nymphal (1^st^ and 2^nd^) instars over the two-week period was highest for tree of heaven and hops (>65%), followed by muscadine grape (<40%) and kiwi (<10%) (**Figure 2A**; χ^2^ = 343, df=3, p < 0.001). Survivorship for later stage nymphs was, again, highest when feeding on tree of heaven, with survivorship on muscadines significantly higher than on either kiwi or hops (**Figure 2B**; χ^2^ = 86.3, df=3, p < 0.001). Later stage nymphs had greater than 75% survivorship on all hosts until day 10, followed by a sharp decline in survival in the last four days. Pre-reproductive adults only survived well on tree of heaven (>90% at 14 d), with steady decline in survival probability when feeding on the other three host species, ending with less than 25% survivorship on day 14 (**Figure 2C**; χ^2^ = 66.5, df=3, p < 0.001). Reproductively mature adult SLF experienced substantial early die-off beginning on Day 4; adults feeding on muscadine grape and hops had <40% survival probability after 4 days. While tree of heaven sustained SLF survivorship well, SLF feeding on the other host plants had significantly lower probability of survival (**Figure 2D**; χ^2^ = 59.8, df=3, p < 0.001).

### Survival and development on winegrape and black walnut supported diets

**Figure 3:**
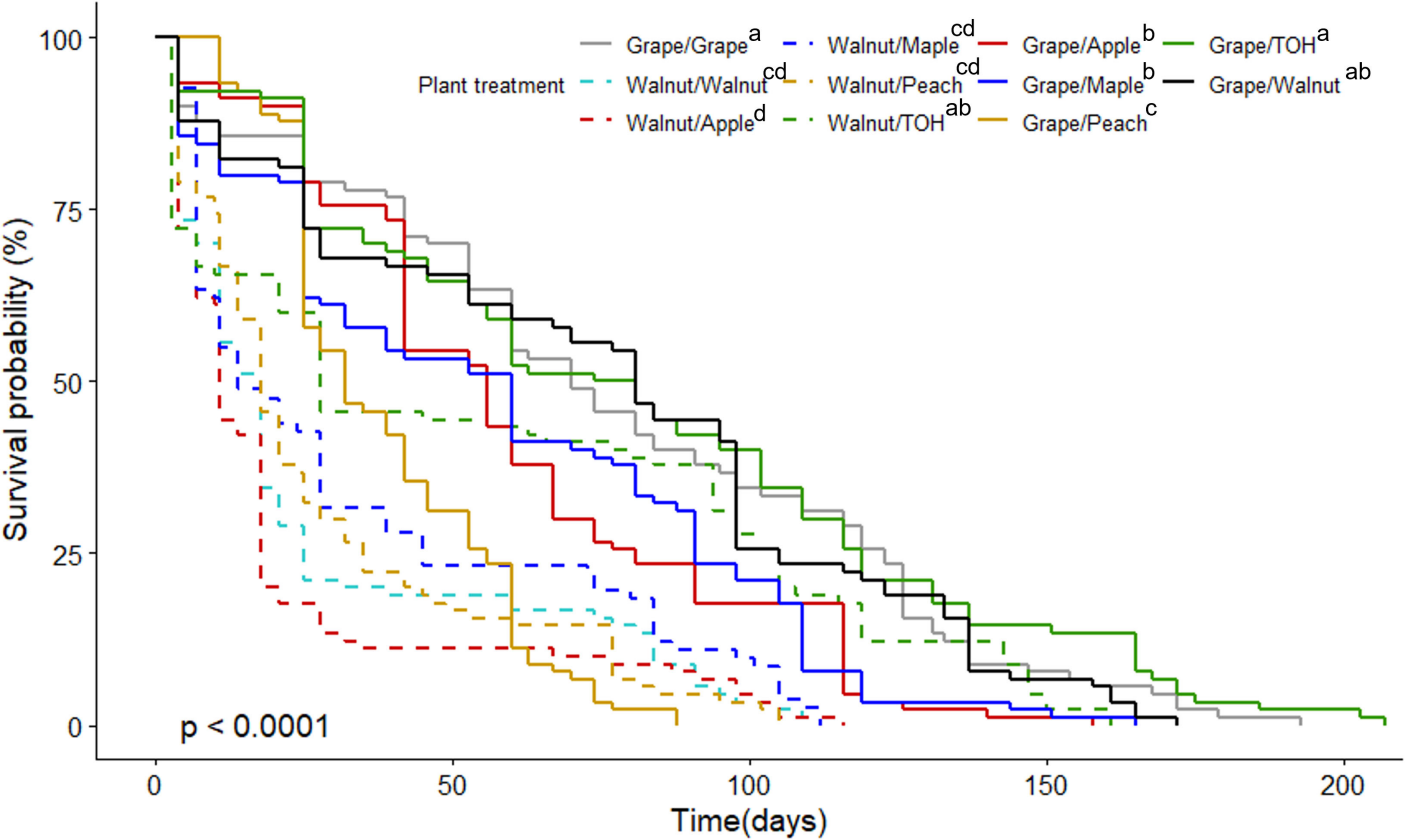
Survival curve of SLF on all treatment combinations using grape and walnut-paired diets. χ^2^ = 279, df = 10, p < 0.0001. Grape diets are depicted with a solid line and walnut diets are dashed. Color of the lines corresponds to the second plant host in the treatment combination. The grape/walnut treatment is a solid black line. Treatments followed by the same letter are not significantly different from one another at α=0.05. TOH = tree of heaven.

All host combinations supported SLF development through to adulthood except winegrape/peach diet where no SLF completed development to the 4^th^ instar stage. Among the other treatments, the four diets of winegrape/winegrape, winegrape/tree of heaven, winegrape/walnut, and walnut/tree of heaven had the highest overall survival probability, which includes time spent both in the nymphal and adult stage (**Figure 3**; χ^2^ = 279, df=10, p < 0.001). These diet treatments had the highest percentage of SLF nymphs surviving to adulthood and lived significantly longer as adults (**Table 1**; F _9, 180_ = 6.74, p < 0.001, ANOVA). Adult SLF fed diets of winegrape only and winegrape/tree of heaven survived over 6 weeks (46.5 ± 5.5 d and 45.2 ± 5.9 d, respectively). While total nymphal development time was a significant factor, there were no pairwise differences among the treatments **Table 1**; (F _9, 180_ = 2.54, p = 0.009, ANOVA **Table 1**). Host diet treatments with the highest SLF survival also had lower total average development times, 88.4 d average versus 92.7 d global average. Host diets with the lowest overall survival, percentage survival to adult, and survival as adults were black walnut-based diets: walnut/peach, walnut/apple, walnut/maple, and walnut/walnut. The proportional hazard analysis was in accordance with the log-rank test in terms of ranking the treatment combinations, so is not shown.

**Table 1:** Development and survival parameters for SLF on single or mixed diet treatments. .

Treatment	Mean nymphal development time (d ± SEM)	Survival to adult (%)	Adult survival (d ± SEM)1
Grape/Grape	90.7 ± 1.6	24	45.2 ± 5.9^ab^
Grape/Apple	96.1 ± 2.2	14	22.9 ± 3.6^bc^
Grape/Maple	92.6 ± 2.9	21	20.6 ± 2.4^c^
Grape/Peach	n/a	0	n/a
Grape/Tree of heaven	88.3 ± 1.0	38	46.5 ± 5.5^a^
Grape/Walnut	87.7 ± 1.9	41	32.1 ± 3.3^abc^
Walnut/Walnut	95.4 ± 4.3	5.6	8.6 ± 3.1^c^
Walnut/Apple	93.8 ± 1.6	6.7	10.0 ± 3.1^c^
Walnut/Maple	101.7 ± 2.0	3.7	4.7 ± 2.33^c^
Walnut/Peach	93.2 ± 4.6	5.6	5.0 ± 1.9^c^
Walnut/Tree of heaven	87.0 ± 1.3	36.7	31.6 ± 4.2^abc^

Single diets included two plants of the same species. Nymphal development time calculated as time required to go from first hatch until adult emergence reported in days (d) ± standard error of the mean (SEM). All SLF in the grape/peach treatment died before completing nymphal development. Development time was significant, but no pairwise differences were observed (F_9,180_ = 2.535, p = 0.009; ANOVA, Tukey HSD). Survival times in the adult survival column followed by the same letter are not significantly different from one another at α=0.05. n/a, not applicable; all SLF in this treatment died before completing all four nymphal stages.

Within each life stage, there was no relationship between instar period and total development time (**Table 2**). In general, SLF that spent a shorter time in the first instar stage spent a longer amount of time in the second instar stage. The same was true for those SLF with longer development periods as first instars had shorter second instar periods. The length of development in the third instar stage was not different among the diet treatments, while the final pre-imago stage was the most variable period, ranging from 22.9-34.9 d, average 26.33 ± 0.35.

**Table 2:** SLF development time within each nymphal life stage for single and mixed diet treatments.

Treatment	Mean time in life stage (d ± SEM)			
	1st Instar	2nd Instar	3rd Instar	4th Instar
Grape/Grape	23.4 ± 0.5^cd^	19.2 ± 0.8^c^	19.7 ± 0.8	28.8 ± 1.9^ab^
Grape/Apple	24.3 ± 0.3^cd^	18.3 ± 0.9^bc^	23.4 ± 1.5	34.9 ± 2.0^b^
Grape/Maple	26.0 ± 0.7^d^	20.9 ± 0.6^c^	21.0 ± 1.3	24.6 ± 2.0^a^
Grape/Peach	25.4 ± 0.5^cd^	13.4 ± 1.5^a^	24.0 ± 1.7	N/A
Grape/Tree of heaven	25.5 ± 0.0^cd^	14.9 ± 0.8^ab^	21.1 ± 0.6	27.3 ± 1.0^a^
Grape/Walnut	23.8 ± 0.4^bc^	19.1 ± 0.7^c^	19.3 ± 0.9	24.2 ± 1.0^a^
Walnut/Walnut	19.0 ± 1.4^a^	20.7 ± 1.6^c^	24.2 ± 2.1	28.4 ± 2.6^ab^
Walnut/Apple	21.1 ± 1.5^abc^	18.3 ± 2.4^abc^	24.8 ± 1.2	26.0 ± 1.5^ab^
Walnut/Maple	19.8 ± 1.9^ab^	22.5 ± 2.4^c^	21.8 ± 1.2	30.7 ± 1.3^ab^
Walnut/Peach	22.4 ± 1.5^abcd^	16.9 ± 1.7^abc^	21.5 ± 4.6	26.2 ± 1.8^ab^
Walnut/Tree of heaven	23.1 ± 0.7^bcd^	19.0 ± 0.7^c^	22.0 ± 0.8	22.9 ± 1.1^a^

Single diets included two plants of the same species. Times within the same column sharing the same letter are not significantly different from one another (1^st^ instar: F_10,522_ = 7.383, p < 0.001; 2^nd^ instar: F_10,402_ = 6.094, p < 0.001; 3^rd^ instar: F_10,298_ = 2.143, p = 0.021; 4^th^ instar: F_9,180_ = 4.281, p < 0.001; ANOVA, Tukey HSD). No pairwise differences for third instar treatments were significant. N/A, not applicable; no SLF in this treatment survived to the fourth nymphal instar stage.

A total of 76 adult female SLF were dissected to assess reproductive development, 18 of which were too degraded to score for some, but not all of the parameters. Of these, only one female (from the winegrape/tree of heaven diet treatment) was mated. The most reproductively developed females occurred in the diets most favorable for nymphal development: winegrape/tree of heaven (Previtellogenic-I: 5 females; Previtellogenic-II: 9; n = 18), walnut/tree of heaven (Previtellogenic-I: 8 females; Previtellogenic-II: 3, Vitellogenic-III: 4; n = 21), winegrape/walnut (Previtellogenic-I: 8 females; Previtellogenic-II: 11; Vitellogenic-III: 1; n = 20) (**Table 3**). No females had fully mature ovaries or oocytes present. Twelve females (18.5%) received a score of (III) for bursa copulatrix development, while seven scored (II), and the remainder scored (I) (**Table 3**). Five males emerged from the walnut only treatment, but no females were available for dissection.

**Table 3:** Reproductive development parameters of adult female SLF.

Treatment	N	No. Females	Mean Lateral Yellow Area (mm^2^)	Ovary Development				Bursa Copulatrix Development				Bursa Copulatrix Sclerotization		
				n/a	I	II	III	n/a	I	II	III	n/a	I	II
Grape/Grape	19	7	0.138	1	–	6	–	1	3	2	1	1	4	2
Grape/Apple	13	2	0.378	–	2	–	–	–	2	–	–	–	2	–
Grape/Maple	18	5	0.036	–	4	1	–	–	1	–	–	–	3	–
Grape/Peach	0	0	–	–	–	–	–	–	–	–	–	–	–	–
Grape/Tree of heaven	35	18	0.305	4	5	9	–	6	6	3	3	7	6	5
Grape/Walnut	41	20	0.437	–	8	11	1	2	13	–	5	2	13	5
Walnut/Walnut	5	0	–	–	–	–	–	–	–	–	–	–	–	–
Walnut/Apple	6	3	0.278	1	1	1	–	1	2	–	–	1	2	–
Walnut/Maple	3	1	0.063	–	1	–	–	–	1	–	–	–	1	–
Walnut/Peach	4	2	0.062	–	2	–	–	–	2	–	–	–	2	–
Walnut/Tree of heaven	31	18	0.207	5	8	3	2	6	9	1	2	7	7	4

‘N’ represents the total number of SLF adults emerging from that treatment, ‘No. Females’ is the number that were female. Only females were dissected. The lateral yellow area describes an area on the side of the abdomen that becomes larger over time and with reproductive maturity. Definitions for the scoring matrix can be found in the Methods section. ' - ' = no female SLF were available to measure, or none were designated in that specific sub-category. n/a, not applicable, the female SLF was not able to be measured for that parameter.

## Discussion

These results confirm and expand the literature on the relationship between SLF fitness and feeding on common specialty crop and wild tree species of the eastern United States. Evaluating SLF survival on three vine specialty crops over two weeks revealed kiwi as an adequate host crop for late instar and early season, pre-reproductive adult survival, while hop plants were as good as tree of heaven for early instar nymph survival. In the development study, SLF had the highest survivorship and fastest development rates on diets of winegrape/winegrape, winegrape/walnut, or either of those species paired with tree of heaven. Spotted lanternfly fed a diet of peach, maple and apple-paired treatments had low rates of survival to adulthood, even when paired with preferred host plant, winegrape. Black walnut diets generally did not support significant development of SLF alone or in combination with a second plant species, unless paired with winegrape or tree of heaven, highlighting the intricacy of SLF nutritional needs. Female reproductive development was positively associated with development and survival parameters. Still, the specific nutritional requirements for SLF growth, development, and reproductive maturity remain elusive.

Total nymphal development length was numerically shorter for higher quality pairings but showed no uniform pattern within each instar stage. In other Hemipteran pest species, host quality plays a significant role in the length of nymphal instar periods and survivorship, in that high quality hosts decrease instar period length (e.g., 35–37), including the invasive *Halyomorpha halys* (Stål) (Heteroptera: Pentatomidae) in a similar single and mixed diet study revealed hosts such as peach provided high survivorship and short developmental times (38). While the total development time of immature SLF was not significantly different among the diet treatments, the number of individuals that survived to adulthood and their subsequent lifespan emphasizes the role of host quality on SLF fitness. Here, SLF longevity and hardiness were compromised when not given access to either tree of heaven or winegrape. The search for hosts providing adequate nutrition may be a primary reason SLF are observed dispersing within and across the landscape (29, 39, 40).

While nutrition is likely a key factor, the outcomes observed here may also be attributed to specific insect and plant physiological features. The kiwi plant used in this study (*Act. deliciosa* ‘Hayward’ grafted onto *Act. chinensis* ‘Tango’) has vine-like growth with pubescent stems and tomentose leaves. First and second SLF instars may not possess a proboscis with the length sufficient to get through the plants’ physical defenses (41). Indeed, later instar nymphs and adults survived better on kiwi, potentially in part due to larger mouthparts. The leaf and stem characteristics of the common kiwifruit, *Act. chinensis*, are glabrous so early instars may be able to exploit vines of the more widely grown kiwi species (42).

Plant size may have also affected survival of SLF adults. Spotted lanternfly spend much of their adult stage feeding and tend to be found feeding on the trunks of trees, unlike nymphs who access phloem from smaller diameter tree limbs and herbaceous plant material (39). These observations suggest that larger, woody plants may yield a greater resource-to-energy expense ratio than herbaceous plants, an advantage only later SLF life stages can utilize. As such, the 30 cm tall, younger plant material used in this greenhouse study may not have contained sufficient phloem volume for the prodigious feeding behavior of adults and affected their survival, though we tried to compensate for this possibility with frequent plant replacements. Although previous greenhouse studies have shown that SLF can reproduce on these smaller trees (8), here, only one female was mated and none had fully developed ovaries despite some adults living in excess of 6 weeks. This may be due in part to the conditions under which these SLF were held. In studies designed to develop a rearing protocol for this invasive species, females reproduced more reliably when provided with an oviposition substrate such as a tree of heaven log and held in a growth chamber at 12L:12D and (24°C:13°C) compared with those held in a greenhouse with natural light and temperatures between 21-25°C (similar to conditions in our experiment) or in a growth chamber at 16L:8D and ~24°C (8). Our experimental design did not ensure equal adult sex ratios, so further research to assess the impact of these diets on SLF reproductive development is needed to clarify questions about mating and reproductive maturity.

Nevertheless, we can contextualize the results of this study to others in this field. Like others, we continue to see low developmental success and survivorship of SLF on apple and peach plants, suggesting the large presence of SLF observed in orchards may be less of a concern than initially thought. Still, researchers in China have reported damage to peach trees by SLF (21, 43), and others recently found that feeding by SLF on young, non-bearing peach trees resulted in increased frost injury the following spring (LJN, *personal observation*). However, as SLF does not survive well on peach based on results of this study and in other studies, these impacts may be rare (11, 28).

Winegrape continues to be a key host for all life stages of SLF. The present study used the common winegrape, *V. vinifera* ‘Riesling’. A similar study assessing the effect of mixed diets on SLF development used a different species of grape, the scuppernong, *V. rotundifolia* (11). Fruits of this species, also called muscadine, are eaten fresh or made into a type of wine. Spotted lanternfly developing on *V. rotundifolia* only completed development to the third instar before dying out (11), similar to the winegrape/peach diet in the current study. SLF reared on *V. vinifera* however could fully develop to adulthood, with some adults living more than 6 weeks. While comprehensive research on the performance of SLF feeding on different *Vitis* spp. has not taken place, it would be warranted due to the documented damage and preference observed for various grape species.

Results from this study add to the building literature that SLF can survive and develop without access to what is often considered their primary or preferred host, tree of heaven. Interestingly, while *V. vinifera* continues to be a good host by itself, combining it with certain species, specifically peach, increased immature mortality and halted development at the third instar stage. The vine species tested could sustain SLF for about a week with low mortality, though survival likelihood declines rapidly in subsequent days. While tree of heaven is a major predictor of suitable habitat, SLF can likely be found persisting in areas without tree of heaven, but with access to winegrape and to a smaller extent black walnut. Some vineyards have begun removing tree of heaven from wooded areas close to their vines to reduce SLF habitat, a strategy that might not be effective if SLF can persist to a high degree on the grape host or if they can develop successfully on other yet unknown wild hosts, providing source populations for dispersal into vulnerable crops such as winegrape.

## Data availability statement

The raw data supporting the conclusions of this article will be made available by the authors, without undue reservation.

## Author contributions

All authors conceived, designed, and conducted the research. JE, SJ, LN, and JU conducted the experiments. JE analyzed the data, conducted statistical analyses, and wrote the initial manuscript. TL and JU secured funding. All authors contributed to the article and approved the submitted version.
